# Women with symptoms of uncomplicated urinary tract infection are often willing to delay antibiotic treatment: a prospective cohort study

**DOI:** 10.1186/1471-2296-14-71

**Published:** 2013-05-31

**Authors:** Bart J Knottnerus, Suzanne E Geerlings, Eric P Moll van Charante, Gerben ter Riet

**Affiliations:** 1 Department of General Practice, Academic Medical Center - University of Amsterdam, PO Box 22700, 1100 DE, Amsterdam, The Netherlands; 2 Department of Internal Medicine / Infectious Diseases, Academic Medical Center - University of Amsterdam, PO Box 22700, 1100 DE, Amsterdam, The Netherlands

**Keywords:** Urinary tract infections, General practice, Antibiotics, Delayed treatment, Natural course

## Abstract

**Background:**

Women presenting with symptoms of acute uncomplicated urinary tract infection (UTI) are often prescribed antibiotics. However, in 25 to 50% of symptomatic women *not* taking antibiotics, symptoms recover spontaneously within one week. It is not known how many women are prepared to delay antibiotic treatment. We investigated how many women presenting with UTI symptoms were willing to delay antibiotic treatment when asked by their general practitioner (GP).

**Methods:**

From 18 April 2006 until 8 October 2008, in a prospective cohort study, patients were recruited in 20 GP practices in and around Amsterdam, the Netherlands. Healthy, non-pregnant women who contacted their GP with painful and/or frequent micturition for no longer than seven days registered their symptoms and collected urine for urinalysis and culture. GPs were requested to ask all patients if they were willing to delay antibiotic treatment, without knowing the result of the culture at that moment. After seven days, patients reported whether their symptoms had improved and whether they had used any antibiotics.

**Results:**

Of 176 women, 137 were asked by their GP to delay antibiotic treatment, of whom 37% (51/137) were willing to delay. After one week, 55% (28/51) of delaying women had not used antibiotics, of whom 71% (20/28) reported clinical improvement or cure. None of the participating women developed pyelonephritis.

**Conclusions:**

More than a third of women with UTI symptoms are willing to delay antibiotic treatment when asked by their GP. The majority of delaying women report spontaneous symptom improvement after one week.

## Background

Acute uncomplicated urinary tract infections (UTIs) are infections of the lower urinary tract in otherwise healthy, non-pregnant, adult women without known anatomical or functional abnormalities of the urinary tract. The symptoms are bothersome and have a negative impact on quality of life [1-4]. Although empiric antibiotic treatment of all women with urinary symptoms has been reported to be cost-effective [5,6], bacterial resistance is rising [7-10] and strategies to reduce antibiotic use are needed.

Placebo arms of randomized trials have shown that 25 to 50% of women presenting with UTI symptoms will have recovered in one week without using antibiotics [11-13]. Moreover, qualitative research has suggested that these women often want to avoid taking antibiotics and may prefer delayed antibiotic treatment [14]. Therefore, antibiotic use might be reduced if all women with UTI were asked to delay treatment.

We investigated how many women with UTI symptoms were willing to delay antibiotic treatment when asked by their general practitioner (GP). In addition, we explored how many of these women reported not to have used antibiotics after one week and whether their symptoms had improved.

## Methods

### Design and setting

From 18 April 2006 until 8 October 2008, in a prospective cohort study, patients were recruited in 20 GP practices in and around Amsterdam, the Netherlands, as part of the Amsterdam Cystitis / Urinary Tract Infection Study (ACUTIS) [15]. These practices serve a population of approximately 47000 people.

### Participants

Eligible were female patients over 12 years of age, contacting their GP with painful and/or frequent micturition. The symptoms had to be present for no longer than seven days.

Exclusion criteria were: pregnancy, lactation, signs of pyelonephritis, having used antibiotics or having undergone a urological procedure in the past two weeks, known anatomical or functional abnormalities of the urogenital tract, and being immunocompromised (with the exception of diabetes mellitus).

### Assessments

Included patients filled in a questionnaire to record presence and severity of signs and symptoms on a 4-point scale, each individual symptom being scored as ‘absent’, ‘a little’, ‘considerable’ or ‘very much’, In addition, they collected a urine sample for urinalysis and culture according to pre-specified criteria [15]. In line with the national guideline of the Dutch College of General Practitioners [16], no instructions for the urine collection method were given, since these have been reported to have no consequences for the extent of contamination [17-19]. The result of the baseline culture became known after the follow-up period of one week, ≥10^3^ colony-forming units (CFU) of a single uropathogen per milliliter (mL) being defined as a positive culture according to international guidelines [20]. If two organisms were grown, a culture was considered positive if ≥10^3^ CFU/mL of a primary uropathogen (*Escherichia coli* or *Staphylococcus saprophyticus*) were found, according to the same guidelines.

After performance of clinical history and urinalysis, GPs were requested to ask all patients whether they were willing to delay antibiotic treatment as long as possible. During this period, participating patients could still change to antibiotic treatment at any time. After one week, patients were asked to report whether they had used any antibiotics (yes/no) and whether their symptoms had been cured, had improved, were unchanged or had deteriorated.

### Analysis

Key characteristics from history and urinalysis were compared between patients who were asked by their GP to delay antibiotic treatment and patients who were not. For patients who were asked to delay, we determined how many were willing to do so and how many were not. We crosstabulated the data on clinical cure and baseline culture in women who were willing to delay antibiotic treatment and had not used any antibiotics during the following week. If no follow-up data on antibiotic use were available, patients were analyzed as having used antibiotics. Analyses were performed in Stata/SE, version 10.1.

### Ethics and informed consent

The study procedure was approved by the Medical Research Ethics Committee of the Academic Medical Center in Amsterdam. Participating women received a letter with information about the study and provided written informed consent. For patients under the age of 18, written parental authorization was obtained.

## Results

**Figure 1:**
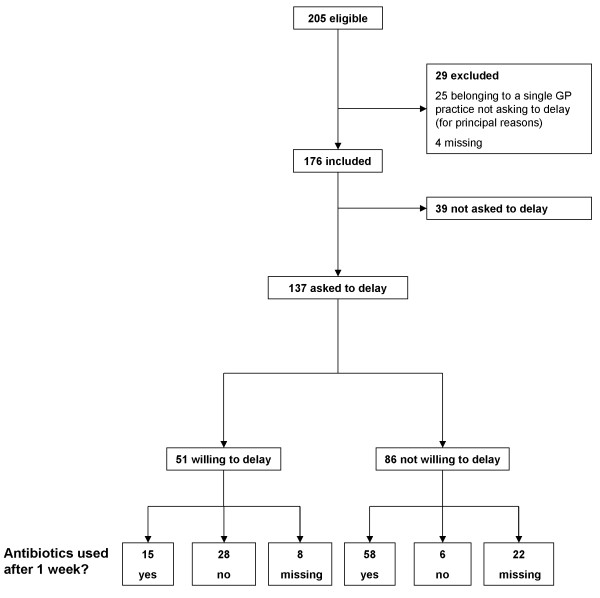
**Flow chart In total, 205 women were eligible.** For principal reasons, one participating GP surgery did not ask any of their 25 patients to delay antibiotic treatment. For four patients, the GP did not report whether they were asked to delay. Of the remaining 176 patients, 137 were asked by their GP to delay antibiotic treatment. Of these patients, 37% (51/137) were willing to delay, of whom 55% (28/51) did not use any antibiotics during the following week.

In total 205 women were eligible, of whom 29 were excluded (Figure 1). GPs participating in one of the 20 GP practices did not ask any of their 25 patients to delay antibiotic treatment, because they disagreed in principle with this approach. No differences in main patient characteristics (age, socioeconomic status, number of diagnosed UTIs in the past) were present between patients of this non-participating practice and those of the other practices. For four patients, GPs did not report whether they had been asked to delay.

Of the remaining 176 patients, 137 were asked by their GP to delay antibiotic treatment and 39 were not. For these two groups, median numbers of recruited patients per practice were 5 (range 0–33) and 1 (range 0–9), respectively. Table 1 shows the main characteristics of the two groups. Overall UTI prevalence was 59% (103/176). Women who reported at least considerable pain and women who thought they had a UTI were more likely to be asked to delay antibiotic treatment. Of those asked, 37% (51/137) were willing to delay (Figure 1). To investigate the variation between practices with respect to the proportions of women willing to delay, we calculated a weighted mean proportion across practices (random-effects meta-analysis), which yielded a similar result (39% (95% CI 27-52%)). Severity of symptoms did not differ significantly between women willing to delay and women not willing to delay (Table 2). The median number of recruited patients per practice was 2 for women willing to delay (range 0–18) and 4 for women not willing to delay (range 0–15).

**Table 1:** Patient characteristics: asked to delay vs not asked to delay

**Characteristic**	**Asked by gp**			**Not asked by gp**			**p value**
	**n**	**(%)**	**missing (n)**	**n**	**(%)**	**missing (n)**	
**Total**	137	(100)		39	(100)		
**History**							
mean age in years (range)	42	(16–89)	3	37	(16–70)	5	0.11
duration of symptoms > 1 day	95	(69)	5	23	(59)	4	0.26
at least considerable frequency	86	(63)	3	19	(49)	4	0.12
at least considerable pain	66	(48)	3	11	(28)	4	0.03
any vaginal irritation	57	(42)	3	15	(38)	5	0.72
at least 1 UTI ever diagnosed	106	(77)	4	27	(69)	5	0.30
patient thinks she has a UTI	122	(89)	3	23	(59)	5	0.00
**Stick**							
nitrite positive	47	(34)	1	12	(31)	1	0.68
blood ≥ 1+	85	(62)	1	27	(69)	0	0.41
leucocytes ≥ trace	114	(83)	1	27	(69)	1	0.05
**Culture**							
culture positive	84	(61)	6	19	(49)	1	0.16

Of the 176 included patients, 137 were asked by their GP to delay antibiotic treatment and 39 were not. Women who reported at least considerable pain and women who thought they had a UTI were more likely to be asked to delay antibiotic treatment.

**Table 2:** Patient characteristics: willing to delay vs not willing to delay

**Characteristic**	**Willing to delay**			**Not willing to delay**			**p value**
	**n**	**(%)**	**missing (n)**	**n**	**(%)**	**missing (n)**	
**Total**	51	(100)		86	(100)		
**History**							
mean age in years (range)	43	(16–79)	1	42	(16–89)	2	0.75
duration of symptoms > 1 day	38	(75)	1	57	(66)	4	0.31
at least considerable frequency	30	(59)	1	56	(65)	2	0.46
at least considerable pain	23	(45)	1	43	(50)	2	0.52
any vaginal irritation	21	(41)	1	36	(42)	2	0.94
at least 1 UTI ever diagnosed	39	(76)	2	67	(78)	2	0.85
patient thinks she has a UTI	42	(82)	1	80	(93)	5	0.05
**Stick**							
nitrite positive	13	(25)	1	34	(40)	0	0.09
blood ≥ 1+	25	(49)	1	60	(70)	0	0.02
leucocytes ≥ trace	38	(75)	1	76	(88)	0	0.04
**Culture**							
culture positive	26	(51)	0	58	(67)	6	0.06

Of the 137 patients who were asked to delay antibiotic treatment, 51 were willing to delay and 86 were not. Women who had haematuria and leukocyturia on urinalyis were less likely to be willing to delay antibiotic treatment. Severity of symptoms did not differ significantly between women willing to delay and women not willing to delay.

After one week, 55% (28/51) of women who were willing to delay reported not to have used antibiotics and 71% (20/28) of these women reported clinical improvement or cure (Table 3). Of these patients, 35% (7/20) had a positive baseline culture. Of the eight women not reporting clinical improvement or cure after one week, two turned out to have had a positive culture at baseline.

**Table 3:** Association between spontaneous cure and baseline culture among women willing to delay antibiotic treatment

	**Cure**	**Improvement**	**No change**	**Deterioration**	**Total**
**Baseline culture positive**	4	3	2	0	9
**Baseline culture negative**	8	5	5	1	19
**Total**	12	8	7	1	28

The table shows the association between clinical cure and baseline culture in women who were willing to delay antibiotic treatment and did not use any antibiotics during the following week. The result of the baseline culture was known only after the follow-up period of one week.

In total, 28 delaying women did not use any antibiotics. Of these women, 20 (71%) reported clinical cure or improvement. Of the eight women who did not report cure or improvement, two turned out to have had a positive baseline culture.

Of the 51 women willing to delay, 15 (29%) had used an antibiotic after one week, of whom all reported clinical improvement or cure and 13 had a positive baseline culture. Eight of the 51 women (16%) did not report on their antibiotic use.

Of women who had been asked by their GP to delay treatment, 25% (34/137) reported not to have used antibiotics after one week, against 15% (6/39) of women who had not been asked, yielding a difference of 10% (95% CI −6 to 21%). Of the six women not being asked to delay, five had cure or improvement of symptoms, of whom one had a positive baseline culture. Of the 137 women who were asked to delay, 30 did not report on antibiotic use after one week (Figure 1), 22 of whom belonged to the group not willing to delay at baseline. Of the 39 women who were not asked to delay, one did not report on antibiotic use.

The result of the baseline culture was known only after the follow-up period of one week. It turned out to be positive for 51% (26/51) of the delaying women and for 67% (58/86) of the non-delaying women (risk difference 16% (95% CI 0-33%)) (Table 2).

No patients developed pyelonephritis during the follow-up period of one week, implying a 95% CI of 0-12% for the 28 delaying women who had not used antibiotics after one week.

## Discussion

Our results suggest that more than a third of women with UTI symptoms are willing to delay antibiotic treatment when asked by their GP. More than half of these women (28/51) will not have used antibiotics after one week, of whom more than 70% (20/28) will have improvement of their symptoms.

The proportion of women reporting the use of antibiotics was 10% lower in the group that had been asked to delay as compared to those that had not been asked. The true reduction may be somewhat higher, since in the group asked to delay 30 patients were analyzed as having used antibiotics because no follow-up data were available. However, it should be taken into account that not all baseline characteristics were similar between these two groups (in the group asked to delay, more women reported at least considerable pain and/or thought they had a UTI).

As far as we know, this is the first study that describes the proportion of women with UTI symptoms that are willing to delay antibiotic treatment. Our findings are consistent with the results from a qualitative study by Leydon et al., which revealed that patients do not always want to use antibiotics, although clinicians often assume that they do [14]. This misinterpretation by clinicians might be illustrated by the fact that not all eligible patients in our study were asked to delay antibiotic treatment by their GP.

In addition to the 28 women willing to delay treatment, six women not willing to delay had not used antibiotics after one week. Although being asked to delay might have stimulated these six women in changing their minds to not using antibiotics, we did not obtain information on their motives. For five of these women, symptoms were cured or improved, one having had a positive baseline culture.

We had no further information on reasons for GPs not to enquire after their patients’ willingness to delay, which may be seen as the main limitation of our study. However, patients who were excluded by their GP did not have worse baseline characteristics than those who were included. On the contrary: women who reported at least considerable pain and women who thought they had a UTI were more likely to be included. This suggests that the GPs’ decisions whether to ask patients to delay or not may be based more on their personal attitude towards antibiotic prescriptions than on patient characteristics, which is in line with the previously mentioned findings of the study by Leydon et al [14]. In addition, patient attitudes (e.g. demanding personality) and previous experiences (e.g. problematic UTI history) might influence the GPs’ decisions.

Another limitation is the large number of missing data in the group of patients who were not willing to delay antibiotic treatment. More than a third of these patients did not report on antibiotic use and symptom improvement after one week. We consider it likely that most of these patients were cured and therefore did not feel inclined to report on follow-up results.

Due to cultural differences, our results might not be completely generalisable to other countries, where women may be less willing to delay antibiotic treatment than in the Netherlands. However, similar patient attitudes towards antibiotic prescriptions have been reported for the United Kingdom and Hong Kong [14,21,22].

Whereas we evaluated improvement of symptoms after a week, we did not obtain follow-up urine samples. Therefore we can not draw any conclusions on microbiological cure.

An uncommon but severe complication of UTIs is pyelonephritis, which may be a reason to treat all women with a suspected UTI. However, placebo arms of randomized trials suggest that cystitis seldom progresses to pyelonephritis [11-13]. Similarly, no women developed pyelonephritis in our study population.

Since in clinical practice the result of the urine culture is not available at the moment that a treatment decision is taken, we included all patients with symptoms, independent of the result of their urine culture. We consider this a more pragmatic approach than including only patients with microbiologically proven UTI.

Our findings imply that antibiotic use might be considerably reduced by simply asking women with UTI symptoms whether they are willing to delay antibiotic treatment. Besides, most of delaying women will have symptom improvement after one week. This is in line with results from a randomized trial by Little et al., in which a treatment strategy of delayed prescription reduced antibiotic use by 20% while yielding the same symptom control when compared to a strategy of immediate antibiotics [23]. A potentially promising strategy being currently under investigation is initial treatment with pain medication instead of immediate antibiotics [24,25].

## Conclusions

Our results support previous findings that women with UTI symptoms may be more receptive to delayed antibiotic prescriptions than is assumed by many clinicians. If all of these women were asked to delay antibiotic treatment, antibiotic use might be substantially reduced without negatively impacting clinical recovery. As a safe alternative to antibiotics (without the risk of bacterial resistance), symptomatic treatment may be offered.

## Abbreviations

UTI: Uncomplicated urinary tract infection; GP: General practitioner; CFU/mL: Colony-forming units per millilitre.

## Competing interests

The authors declare that they have no competing interests.

## Authors’ contributions

All authors participated in the design of the study. BJK coordinated data collection, statistical analysis and reporting of the results. All authors contributed to writing the paper. All authors have read and approved the final manuscript.

## Pre-publication history

The pre-publication history for this paper can be accessed here:

http://www.biomedcentral.com/1471-2296/14/71/prepub
